# Looking Back to the Future of Mitochondrial Research

**DOI:** 10.3389/fphys.2021.682467

**Published:** 2021-04-30

**Authors:** Paolo Bernardi

**Affiliations:** Department of Biomedical Sciences, University of Padova, Padova, Italy

**Keywords:** mitochondria, ATP synthase, channels, pathophysiology, hypoxia

Ἦθος ἀνθρώπῳ δαίμων–*ethos anthropoi daimon*—is a famous aphorism of the Greek philosopher Heraclitus (544–483 BC). While its deeper meaning is probably more complex, the conventional translation is “a human being's character is his/her fate.” When I was asked by George Billman to contribute my thoughts on the future of mitochondrial research it occurred to me that perhaps I could try to foretell the fate of mitochondrial research from its character, i.e., from the key themes from which the discipline developed. I will limit this brief comment to a few topics that also reflect my own interests, and that should not be considered even an attempt to be exhaustive. In the twentieth century the key issue in Bioenergetics (hence in mitochondrial research) has been the mechanism of energy conservation. The turning point was the proposal and then the demonstration of Peter Mitchell's chemiosmotic hypothesis, i.e., that in mitochondria the basic events are the coupling of aerobic electron transfer to H^+^ pumping, the formation of the H^+^ electrochemical gradient and its harnessing by the ATP synthase (Mitchell, 1966), reprinted in Mitchell (2011). It is remarkable that the most recent advances in structural biology and superresolution microscopy, which are removing hurdles and moving the boundaries of Science beyond imagination, have confirmed the basic tenets of chemiosmotic principles in amazing detail.

## ATP Synthase

One example of how structural biology complemented physiology is the solution to the question of the H^+^/ATP stoichiometry, which had been the matter of considerable discussion (Brand and Lehninger, 1977; Sholtz et al., 1983). The demonstration that different organisms possess c rings (the rotating barrel powered by H^+^ flux) with a number of subunits varying between a minimum of 8 and a maximum of 17, and the fact that one full rotation cycle generates 3 ATP molecules (Boyer, 1997) has both defined the precise stoichiometry for each type of c ring (between 2.67 and 5.67 H^+^/ATP) and explained the apparent variability in the stoichiometry itself, see Nirody et al. (2020) for a review. While the basic structure and catalytic mechanism of ATP synthases is highly conserved across species, what is most puzzling is the existence of profound differences in the non-catalytic parts of the enzyme, which evolved to include a numer of subunits that were indeed defined “supernumerary” (Vaillier et al., 1999) because they are not essential for the catalytic activity. These subunits are involved in the process of dimerization and of membrane bending that contributes to generate the inner membrane cristae, which are then stabilized by the lateral association of dimers (Paumard et al., 2002; Dudkina et al., 2006; Jiko et al., 2015; Kühlbrandt, 2019; Spikes et al., 2021). And yet, clear species-specific differences exist in primary structure of these subunits, suggesting that they may serve additional function(s) that have not been fully discovered yet (Kühlbrandt, 2019). I think that research on the physiological function(s) of “supernumerary” subunits of ATP synthases (which may include the controversial formation of the high-conductance permeability transition pore) (Giorgio et al., 2013; Alavian et al., 2014; He et al., 2017a,b; Carroll et al., 2019; Mnatsakanyan et al., 2019; Urbani et al., 2019; Pinke et al., 2020) will be a very fruitful field of investigation in the years to come.

## A Supercomplex Matter

High-resolution definition of individual mitochondrial respiratory complexes, which begun over 20 years ago, has also yielded key information on the mechanisms of proton translocation coupled to electron transfer (Tsukihara et al., 1996; Xia et al., 1997; Michel et al., 1998; Baradaran et al., 2013; Zickermann et al., 2015; Kampjut and Sazanov, 2020). How electrons are transferred *between* respiratory complexes and whether this requires stable interactions is a question of great relevance, that to the best of my knowledge was clearly posed and addressed with inhibitor titration studies by Stoner (1984). The existence of respiratory supercomplexes made by associations of complexes I, III, and IV with defined stoichiometries in the native membrane is now established, and the role of supercomplexes in pathophysiology is increasingly appreciated (Schägger and Pfeiffer, 2000, 2001; Bianchi et al., 2004; Acín-Pérez et al., 2008; Dudkina et al., 2011; Lapuente-Brun et al., 2013; Letts et al., 2016; Milenkovic et al., 2017; Rathore et al., 2019; Berndtsson et al., 2020; Protasoni et al., 2020). An important question is the role of the supercomplex assembly factor SCAF1, which has been shown to promote supercomplex formation between CIII and IV (III_2_IV) and contribute to “branching” of the respiratory chain (Calvo et al., 2020). This topic is generating a very lively discussion that is likely to continue in the future (Mourier et al., 2014; Enríquez, 2016; Milenkovic et al., 2017; Lobo-Jarne et al., 2018; García-Poyatos et al., 2020; Protasoni et al., 2020; Fernández-Vizarra et al., 2021).

## DE(Localized) Gradients?

Another interesting question is whether H^+^ pumping (together with the low permeability of the inner membrane) can generate a *delocalized* electric field rapidly spreading to the whole network (Amchenkova et al., 1988), or rather mitochondria should be seen as a mosaic of *localized* coupling units where the H^+^ pumping complexes and ATP synthases are closely spaced to make individual functional units without the need for lateral diffusion of charges (Yaguzhinsky et al., 2006), two hypotheses that may actually not be mutually exclusive (Westerhoff et al., 1986). Recent work using high resolution microscopy has demonstrated that both have merit and that, to some extent, the issue may be a matter of definition (i.e., what is meant by “short” and “long” range) and possibly of anatomy. Long range diffusion may predominate in tissues were mitochondria are mostly in the form of tubular, continuous structures (like in muscle) (Glancy et al., 2015) while local coupling (with formation of individual disc-shaped crista structures) may prevail where (or when) mitochondria are in the form of individual organelles (Wolf et al., 2019). This area of research has also made great progress on the fusion-fission events that regulate mitochondrial morphology and function (Giacomello et al., 2020). I think that superresolution microscopy together with the genetic manipulation of determinants of mitochondrial morphology will allow further definition of subcellular electrical events that bear both on mitochondrial function and on the shaping of localized ion gradients.

## Mitochondrial Dynamics

Mitochondrial dynamics is a topic of enormous interest and of great additional potential in spite of the major progress made in recent years. The pioneering work of Jürgen Bereiter-Hahn provided a detailed description of mitochondrial motion and of fusion-fission events *in vivo* (Bereiter-Hahn, 1990; Bereiter-Hahn and Voth, 1994). The molecular basis for mitochondrial dynamics is being unraveled at a steady pace, and is revealing the delicate balance between proteins that favor mitochondrial fusion and those that promote mitochondrial fission, their relationships with the cell cycle and with mitochondrial responses to pathophysiological perturbations (which depend on the cell type as much as on the stimulus), their role in cell survival and death, and their relationship to proteins that determine the maintenance of mitochondrial ultrastructure and its close interactions with the endoplasmic reticulum (Osteryoung and Nunnari, 2003; Mishra and Chan, 2016; Eisner et al., 2018; Giacomello et al., 2020).

## Cation Channels

When thinking of mitochondria and subcellular, localized ion gradients one obviously thinks of Ca^2+^ (Rizzuto et al., 1993) and this takes us to one of the paradoxes that accompanied the progressive acceptance of the chemiosmotic hypothesis. The existence of a proton electrochemical gradient as the energetic intermediate between respiration and ATP synthesis poses some constraints on membrane permeability to cations. Indeed, it was noted that equilibrium distribution of K^+^ and Ca^2+^ across a membrane maintaining an electrical potential difference of 180 mV (negative inside) would have meant matrix concentrations of about 150 M for K^+^ and 1 M for Ca^2+^, see Bernardi (1999) for a detailed review. Mitchell was aware of this problem and conceived two complementary postulates to solve it. The first (3rd postulate of chemiosmosis) is that the inner membrane possesses electroneutral H^+^-cation exchangers allowing extrusion of cations entering the matrix down their electrochemical gradient. Operation of the exchangers (Mitchell and Moyle, 1969; Garlid, 1978, 1979) prevents the otherwise inevitable accumulation of cations that would lead to swelling and osmotic lysis of the organelle. The quest for the K^+^-H^+^ exchanger is still under way, although the LETM1 protein is clearly involved in mitochondrial cation homeostasis through modulation of the K^+^-H^+^ exchange process (Nowikovsky et al., 2004, 2007) and possibly also of electroneutral Ca^2+^-H^+^ exchange (Tsai et al., 2014), an issue that is still the matter of discussion (De Marchi et al., 2014; Nowikovsky and Bernardi, 2014), see Austin and Nowikovsky (2019) for a recent review. The second (4th postulate of chemiosmosis) is that the inner membrane has a low permeability to protons and to anions and cations generally (Mitchell, 1966, 2011). The latter point was almost universally (and as it turns out, erroneously) taken to mean that mitochondria could not possess channels for cations, a point that pervaded the literature well until the turn of last century (Garlid et al., 1989). This state of affairs considerably delayed the discovery of the mitochondrial Ca^2+^ uniporter (MCU) and its regulatory subunits (Perocchi et al., 2010; Baughman et al., 2011; De Stefani et al., 2011; Mallilankaraman et al., 2012; Sancak et al., 2013; Kamer and Mootha, 2014; Mammucari et al., 2016) and the assessment of their role in disease (Logan et al., 2014; Debattisti et al., 2019); and of mitochondrial K^+^ channels (Inoue et al., 1991; Szewczyk et al., 2006; Szabó and Zoratti, 2014; Paggio et al., 2019). The great wave coming from these areas of research is unlikely to subside, and will translate in more breakthroughs on how mitochondrial participate and contribute to the shaping of intracellular ion gradients.

## Inner Membrane Permeability and Pathophysiology

As the chemiosmotic hypothesis became consolidated, a set of early observations on the Ca^2+^-dependent permeability increase to ions and solutes through “permeability defects” with a pore radius of 14 Å (Massari and Azzone, 1972) became widely interpreted as an *in vitro* artifact of little relevance to mitochondrial physiology, see Bernardi et al. (2006) for a specific review. Only a few Authors interpreted the increased permeability (defined permeability transition, PT, by Haworth and Hunter) as a potentially regulated event serving a role in pathophysiology (Haworth and Hunter, 1979; Hunter and Haworth, 1979a,b; Pfeiffer et al., 1979; Crompton et al., 1987), possibly as a regulated pathway for Ca^2+^ release (Bernardi and Petronilli, 1996), which is consistent with a number of observations (Carraro et al., 2020). While today there is a general agreement that the PT is mediated by opening of a channel, its molecular identity is the matter of discussion. The latest results suggest that the PT can be mediated by a Ca^2+^-dependent conformational change of both the adenine nucleotide translocator (ANT) and the ATP synthase, through mechanisms that still need to be defined, see Carraro et al. (2020) for a discussion. The PT has been shown to play a role in necrotic cell death in a set of studies (Duchen et al., 1993; Imberti et al., 1993; Pastorino et al., 1993) that were greatly helped by the demonstration that the PT is inhibited by cyclosporin A (Fournier et al., 1987; Crompton et al., 1988; Broekemeier et al., 1989) through the matrix protein cyclophilin D (Halestrap and Davidson, 1990; Nicolli et al., 1996). The PT was then shown to play a role in apoptosis as well (Marchetti et al., 1996). Together with the discovery that cytochrome c release from the intermembrane space triggers the mitochondrial pathway of apoptosis through activation of procaspase 9 (Liu et al., 1996), these studies opened a new season in mitochondrial research that is lasting to this day for its major implications in the pathogenesis of both degenerative diseases and cancer. Selective cytochome c release can be achieved by Bax/Bak-dependent permeabilization of the outer mitochondrial membrane following insertion of tBid generated by activation of caspase 8 (Wei et al., 2000) in a process that is substantially opposed by the antiapoptotic protein Bcl-2 (Susin et al., 1996; Yang et al., 1997). Release of cytochrome c can also be a consequence of PTP-dependent swelling (Petronilli et al., 1994) and/or cristae remodeling (Scorrano et al., 2002), and there is an intriguing promoting effect of Bax/Bak (Karch et al., 2013) and an inhibitory effect of Bcl-2 on onset of the PT (Susin et al., 1996). The latter is contrasted by Bcl-2 small molecule interactors (Milanesi et al., 2006) able to reactivate the mitochondrial death program (Oltersdorf et al., 2005) and these regulatory events extend to a variety of Bcl-2 family members (Singh et al., 2019). In a striking therapeutic development, the Bcl-2 ligand ABT-199 (venetoclax) has been introduced in the treatment of a variety of hematologic malignancies (Souers et al., 2013; de Ridder et al., 2021). It should also be mentioned that mitochondria play a key role in degenerative diseases, particularly muscular dystrophies and neurodegenerative conditions ranging from Parkinson's to Alzheimer diseases, amyotrophic lateral sclerosis, multiple sclerosis; and in organ ischemia-reperfusion injury. The mechanisms and targets, which include the PT, are so many that I will not even try to list them, but I would like to mention early work that anticipated these modern developments of mitochondrial pathophysiology (Hunter and Ford, 1955; Kasbekar and Sreenivasan, 1956; Hoch, 1962; Luft et al., 1962; Wollenberger et al., 1963; van Wijngaardeen et al., 1967; Sternlieb, 1968; Jennings et al., 1969; Fleckenstein et al., 1974; Wrogemann and Pena, 1976; Singer et al., 1987), see Bernardi et al. (2015) for relevant literature.

## Adenine Nucleotide Translocator and Uncoupling Proteins

Another historical area of research where breakthroughs are being made is that of nucleotide transport via the ANT. It had long been proposed that the overall exchange of ADP for ATP was mediated by a single substrate-binding site alternately accessible from either side of the membrane (Klingenberg, 1979; Ruprecht et al., 2014). The most recent structures fully confirm this single-pore gating mechanism, whereby in energized mitochondria the nucleotide exchange reaction is mediated by unidirectional uptake of ADP and efflux of ATP “taking turns” on the carrier (Ruprecht et al., 2019). Many issues still await an answer, however. It has recently been shown that in the presence of arachidonic, palmitic or lauric acid the ANT can also transfer H^+^ in mitochondria that do not express uncoupling protein 1 (UCP1) (Bertholet et al., 2019), the bona fide H^+^ channel that mediates non-shivering thermogenesis in brown fat (Nicholls, 1976; Rafael and Heldt, 1976). The existence of ANT-mediated H^+^ currents detected in patch-clamp experiments (Bertholet et al., 2019) supports the earlier suggestion that the ANT mediates a sizeable fraction of the “H^+^ leaks” responsible for basal respiration (Andreyev et al., 1988; Brustovetsky and Klingenberg, 1994). ANT and UCP1 are closely related proteins and both require long-chain fatty acids for H^+^ translocation and yet the molecular mechanisms appears to differ, as only in UCP1 the fatty acid anion participates in the actual mechanism of H^+^ transport (Fedorenko et al., 2012) while it plays a cofactor role in ANT (Bertholet et al., 2019), see Bernardi (2019) for a summary. It will be interesting to test whether other members of the SLC25 superfamily of mitochondrial solute carriers (Palmieri and Monné, 2016) can mediate the occurrence of H^+^ leaks. An additional open question about the ANT is how it can be transformed by Ca^2+^ in a high-conductance channel stimulated by cyclophilin D with an effect prevented by the cognate inhibitor of the latter cyclosporin A (Brustovetsky and Klingenberg, 1996; Brustovetsky et al., 2002), see Carraro et al. (2020) for a recent discussion.

## Mitochondrial DNA

Mitochondria possess their own DNA and translation machinery. Diseases of mtDNA have first been described not so long ago (Wallace et al., 1988), and a new frontier is the manipulation of mtDNA, which holds great promise for a future correction of mtDNA diseases (Gammage et al., 2018) and possibly to treat cancer (Bonekamp et al., 2020). It is remarkable that only 13 out of the roughly 1,100 proteins found in mitochondria are encoded by mtDNA (Rath et al., 2020). During evolution mtDNA has retained only a core set of genes of the respiratory chain and F-ATP synthase, possibly to permit rapid adaptation to changing environments (Wallace, 2007). How mitochondrial and nuclear genomes integrate in mitochondrial biogenesis remains a fascinating topic (Becker et al., 2019) as is the somewhat specular issue of how cells exploit mitochondrial “diversity” by releasing into the circulation mitochondrial damage-associated molecular patterns (including mtDNA) to engage toll-like receptors and innate immune pathways (Zhang et al., 2010; Shintani et al., 2014; Rodríguez-Nuevo et al., 2018) and activate inflammation (Zhou et al., 2011; Oka et al., 2012; Zhong et al., 2018). This is strikingly similar to the effect of microbial pathogen-associated molecular patterns and provides an exciting link to STING, which regulates the type I interferon response (Sliter et al., 2018). The mechanism for mtDNA release is an interesting issue on its own, because it could be a regulated process mediated by the permeability transition pore (Yu et al., 2020) rather than the unspecific result of cell damage, an issue that will certainly attract more attention.

## An Unexpected Twist on Hypoxia

The discovery that mitochondria are involved in the HIF-mediated response to hypoxia (Samanta and Semenza, 2018) through succinate-dependent stabilization of HIF-1α (Selak et al., 2005) and modulation of expression of cytochrome oxidase subunits (Fukuda et al., 2007) was a turning point for our understanding of metabolic adaptation of tumors, first proposed as a causative event in cancer by Otto Warburg (Warburg et al., 1927; Warburg, 1956). A further mechanistic link was provided by the demonstration that TRAP1, a protein targeted to mitochondria in many tumors, inhibits succinate dehydrogenase and leads to succinate accumulation, stabilizing HIF-1α under normoxic conditions and thus making tumor cells ready to resist the impending onset of hypoxia (Sciacovelli et al., 2013). The unexpected twist is that hypoxia has a *beneficial* effect in disorders of the respiratory chain through activation of an endogenous program that allows adaptation. Chronic hypoxia led to a marked improvement in survival and vital parameters in a mouse model of Leigh syndrome, an effect that could not be explained by activation of the HIF transcriptional program (Jain et al., 2016). Rather, mice underwent an age-dependent decline in overall oxygen consumption with brain hyperoxia, which was normalized by hypoxic breathing, carbon monoxide or severe anemia with matching reversal of the neurological disease (Jain et al., 2019). These exciting new results suggest that unused oxygen rather than hypoxia itself may be the culprit, and open up new perspectives to normalize brain tissue hyperoxia (Jain et al., 2019). Genome-wide CRISPR screens at low oxygen tension have now identified genes with relative fitness defects in high or low oxygen, and most of these did not have an obvious connection to HIF (Jain et al., 2020). Remarkably, knockouts of mitochondrial pathways that are presumed to be essential, including complex I, grew relatively well at low oxygen (Jain et al., 2020). This approach is leading to the discovery of hundreds of genes linked to oxygen homeostasis, and there is more. Hypoxia has recently been shown to induce matrix acidification with release of Ca^2+^ from calcium phosphate precipitates, increased free [Ca^2+^] and matrix influx of Na^+^ on the Na^+^/Ca^2+^ exchanger (Hernansanz-Agustín et al., 2020). Na^+^ interaction with phospholipids then reduced inner membrane fluidity, selectively decreasing mobility of free ubiquinone between complex II and III but not inside supercomplexes, thus leading to increased superoxide production at complex III, a novel control mechanism of redox signaling that may have profound consequences for cellular metabolism (Hernansanz-Agustín et al., 2020).

## Intraorganelle Buffering

Another topic that I find particularly fascinating is intramitochondrial communication between the two “arms” of oxidative phosphorylation, i.e., the respiratory chain and the ATP synthase. Respiratory complex III is assembled from a core containing cytochrome b (the only component encoded by mtDNA) and subunits Qcr7 and Qcr8, followed by the incorporation of all other subunits (Smith et al., 2012). As is the case with other respiratory complexes, specific proteins are required for the assembly of complex III including Bcs1 (Nobrega et al., 1992), an assembly factor that is the most frequent target of mutations in human complex III-related diseases. Extragenic compensatory mutations of yeast *bcs1* have been identified that preferentially target the ATP synthase complex, leading to selective decrease of its ATP hydrolytic activity with substantial preservation of ATP synthesis (Ostojic et al., 2013). Thus, the bioenergetics consequences of respiratory impairment appear to be limited by minimizing the hydrolysis of ATP. These results have recently been extended in a thorough study of the Mootha laboratory, who have found that the cellular defects derived from chemical inhibition of complex V with oligomycin are suppressed by loss of complex I activity induced by both genetic and pharmacological means (To et al., 2019). This is a striking example of “intra-organelle” buffering that was also seen for a variety of other mitochondrial inhibitors, suggesting that certain forms of mitochondrial dysfunction may be buffered with “second site” inhibition within the organelle (To et al., 2019). Consistent with the existence of a regulatory feedback between biogenesis of respiratory complexes and of the ATP synthase, ablation of specific subunits of ATP synthase (that largely prevented its assembly) caused a striking decrease of electron transfer chain complexes, with reduction of respiration to negligible rates (He et al., 2017a,b; Carroll et al., 2019).

## Not All Could be Predicted

The more I tried to cover new perspectives that are rooted in the history of mitochondrial research, the more I realized that my selective account was inevitably leaving out a number exciting developments. I will mention the relationships of autophagy with mitochondrial fission-fusion events (Twig et al., 2008; Lazarou et al., 2015; Dorn, 2016); the role of mitochondria in the antiviral response (Kozaki et al., 2017), in the growth of intracellular parasites (Pernas et al., 2018), in antigen presentation (Matheoud et al., 2016), in T cell function (Okoye et al., 2015; Weinberg et al., 2019) and dysfunction (Desdin-Mico et al., 2020), in metabolic reprogramming of macrophages (Mills et al., 2016; Acín-Pérez et al., 2020), in angiogenesis (Herkenne et al., 2020), in systemic stress response mediated by FGF21 (Forsstrom et al., 2019), in non-alcoholic steatohepatitis, where downregulation of mitochondrial circular RNA prevents inhibition of the permeability transition pore by the SCAR protein (Zhao et al., 2020); recent advances on the mechanism of germline selection of human mtDNA (Wei et al., 2019); and the most unexpected finding that the protein product of the *ARHGAP11B* gene, which plays an essential role in development of the human neocortex (Heide et al., 2020), localizes to mitochondria to inhibit the permeability transition pore (Namba et al., 2020). It is reassuring, indeed, that not all could be predicted.

## Author Contributions

The author confirms being the sole contributor of this work and has approved it for publication.

## Conflict of Interest

The author declares that the research was conducted in the absence of any commercial or financial relationships that could be construed as a potential conflict of interest.
